# Transcriptome Analysis Revealed Unique Genes as Targets for the Anti-inflammatory Action of Activated Protein C in Human Macrophages

**DOI:** 10.1371/journal.pone.0015352

**Published:** 2010-10-15

**Authors:** Claudia P. Pereira, Esther B. Bachli, Dominik J. Schaer, Gabriele Schoedon

**Affiliations:** 1 Inflammation Research Unit, Division of Internal Medicine, University Hospital of Zurich and Zurich Center for Integrative Human Physiology ZIHP, University of Zurich, Zurich, Switzerland; 2 Medical Clinic, Uster Hospital, Uster, Switzerland; Charité-Universitätsmedizin Berlin, Germany

## Abstract

**Background:**

Activated protein C (APC) has been introduced as a therapeutic agent for treatment of patients with severe sepsis due to its unique anticoagulant and anti-inflammatory properties in the vascular system. In this study we investigated novel targets for the anti-inflammatory action of APC in human macrophages.

**Methods:**

Using a genome-wide approach, effects of APC on the expression profile in inflammatory activated human macrophages were analyzed.

**Results:**

We identified, for the first time, genes that are specifically regulated by APC under inflammatory conditions, such as chromatin binding protein 4B (CHMP4B) and p300/CBP-associated factor (PCAF), thus indicating a role of APC in the epigenetic control of gene transcription. A functional assay showed the influence of APC in the acetyltransferase/deacetylase activity of nuclear extracts from inflamed macrophages.

**Conclusion:**

Our data sheds new light on APC targets in inflammation and opens new lines of investigation that may be explored in order to further elucidate its unique molecule properties.

## Introduction

The process of inflammation in response to injury or infection, a crucial part of innate immunity, is characterised by activation, adhesion and transmigration of leukocytes through the vascular endothelium to the site of inflammation with the aim of locally repelling microbial invasion and assisting in the repair of damaged tissues. In inflammation, immunovascular communication of circulating cells and vascular endothelium is mediated by production of pro-inflammatory factors such oxidation products, nitric oxide, inflammatory cytokines and chemokines, and the expression of adhesion molecules [1]. Although local inflammatory processes as a defence mechanism are necessarily transient in nature, requiring a rapid induction and a rapid termination, in severe infection they have significant potential to spread from the local site to non-affected tissues and elicit a systemic inflammatory response. Thus, a severe systemic inflammatory response proceeds to generalised intravascular inflammation together with shock, organ dysfunction and high mortality [2].

Monoytes and macrophages are an essential part of innate immunity. Monocytes can differentiate into dendritic cells and macrophages and are crucial for the initiation of an adaptive immune response, clearance of infectious agents as well as resolution of inflammation. Inflammation due to tissue damage or infection results in macrophage activation so increasing the production of cytokines, chemokines and other inflammatory mediators. The classical activation of macrophages is achieved by stimulation with interferon-γ (INFγ) followed by exposure to a microbial trigger like lipopolysaccharide (LPS) resulting in a pro-inflammatory phenotype [1], [3]. The release of an array of pro-inflammatory mediators that communicate with cells of the vasculature plays a key role in the onset of systemic inflammatory responses. Because of the redundancy of inflammatory mediators involved, therapeutic intervention in severe systemic inflammatory response syndrome is challenging. Targeting the major inflammatory cytokines IL-1 or TNF-α, and lipopolysaccharide (LPS) has thus far been unsuccessful in the treatment of sepsis. Furthermore, during severe systemic inflammation, disseminated intravascular coagulation occurs because of the pro-adhesive endothelial environment and tissue factor expression on monocytes with consecutive intravascular thrombin formation [3], [4].

Activated protein C (APC), a plasma serine protease, is best known for its ability to control thrombin formation. APC acts as an anticoagulant by inhibiting activated clotting factors such as FVa and FVIIIa, thereby attenuating thrombin formation. In addition to its anticoagulant properties, APC has been shown to modulate certain cell functions including inflammation, apoptosis, and vascular permeability [5]. In a previous study, we found that APC affected the pro-adhesive endothelial environment by downregulating the induction of endothelial adhesion molecules and monocyte chemotactic protein-1 [6]. Recombinant human APC has been introduced as a therapeutic agent for treatment of patients with severe sepsis due to its unique anticoagulant and anti-inflammatory properties [7]. However, the exact mechanism of the anti-inflammatory action of APC, in particular on inflammatory activated macrophages, is not fully understood. The anticoagulant effects of APC are due to its binding to endothelial receptors such as thrombomodulin or EPCR [8]. For the anti-inflammtory effects, APC interaction with PAR receptors has been postulated [8], [9], [10]. However, in our model of inflamed human monocyte derived macrophages, the PAR-1 pathway seemed not to be involved in APC mediated changes of cell functions [11]. The precise mechanism of cellular activation by APC hast to be further elucidated.

Genome-wide approaches provide the ability to survey the expression level of thousands of genes simultaneously and are powerful tools for exploring complex interactive networks of genes and signaling pathways. Because macrophages constitute an important cellular compartment involved in severe systemic inflammatory responses and to characterize novel targets for the anti-inflammatory action of the physiologic anticoagulant APC in this cellular system, we undertook expanded gene expression analysis of the whole human transcriptome. Previously, we described the inflammatory Wnt5A pathway as a novel target for the anti-inflammatory action of APC as well as for IL-10, known for it's anti-inflammatory potential in macrophages [12]. In our present study we used the same highly standardised setting of primary human monocyte derived macrophages activated with LPS and IFNγ and whole genome oligonucleotide arrays to define the complete transcriptome of macrophages in response to inflammatory activation. Moreover, by anlysing gene expression data in context of biological functions we defined for the first time the specific expression profile in response to APC in inflammatory activated macrophages. Thereby, we identified as targets of APC signalling, molecules and transcription factors not known to be expressed in macrophages or assigned to inflammatory responses so far. The results presented herein describe for the first time a regulation of chromatin binding protein 4B (CHMP4B), and p300/CBP-associated factor (PCAF) by APC in inflamed macrophages, suggesting a novel role of APC in the epigenetic control of gene transcription through chromatin remodeling by histone acteylation/deacetylation.

## Material and Methods

### Ethics statement

Human monocyte derived macrophages were prepared from buffy coats of healthy blood donors (blood donation program; Swiss Red Cross, Zurich, Switzerland). Informed written consent was obtained from all blood donors at the Zurich Blood Doning Center (Blutspende Zürich, Rütistrasse 19, 8952 Schlieren, Switzerland) according to the guidelines of the Ethics Committee of the Government of Zurich, Kantonale Ethikkommission Zürich (KEK), Sonneggstrasse 12, CH-8091 Zürich, Switzerland.

### Cell culture

Human monocyte derived macrophages were prepared from buffy coats of healthy blood donors as previously described [12]. Briefly, after separation by Ficoll gradient (Ficoll-paque™ Plus, Amersham Biosciences, Amersham Basel, Switzerland) and three washes with Mg/Ca-free phosphate-buffered saline (PBS, Gibco Europe, Basel, Switzerland), cells were suspended in Iscove's modified Dulbecco's medium (IMDM; Invitrogen) supplemented with 10% heat-inactivated pooled human serum (human serum Off The Clott, PAA, Austria) and seeded at a density of 1×10^7^ cells/ml in 6-well tissue culture plates (Falcon Oxnard, USA). Purified monocytes were obtained after 2 h incubation under cell culture conditions in a SteriCult tissue culture incubator (Forma Scientific, Waltham, Massachusetts, USA) and 4 times washing in prewarmed Geys Balanced Salt solution (Gibco) by adherence (purity of >98% as determined by Giemsa staining). After 24 h medium was replaced by IMDM supplemented with 2% human pooled serum. Cells were stimulated for 8 h with human recombinant IFN-γ (100 U/ml), IL-10 (5 ng/ml, both from PrepoTech, Rocky Hill, USA), lipopolysaccharide (LPS 10 ng/mL, from Escherichia coli 055:B5, Difco Laboratories, Detroit, MI, USA), human recombinant APC, 90 nmol/L (5 ug/mL Xigris™, Lilly, Switzerland).

### DNA microarray hybridization and analysis

Differential gene expression profiling of human macrophages was performed by competitive dual color hybridisation on human whole genome 60-mer oligonucleotide microarrays as described previously [12]. The complete set of microarray data is deposited in the ArrayExpress database, accession number E-MEXP-927.

### Functional clustering

To analyze the microarray data in the context of biological functions we used information available from the Gene Ontology (GO) consortium (http://www.geneontology.org). The GO terms represent a defined vocabulary describing the biological process, cellular components, and molecular functions of genes in a hierarchical acyclic graph structure. Statistical analysis was performed using GeneGO software. For each of the existing GO terms, the cumulative number of genes meeting our criteria (e.g. up- or downregulated) of all genes represented in the microarray was calculated. The Z score is calculated for every GO term as described [13]. A positive Z score indicates that there are more genes meeting the criterion in the specific GO term than expected by chance. The Z score is transferred to p-values under the assumption of a hypergeometric distribution.

### RNA isolation and quantitative real-time reverse transcription PCR (RT-PCR)

Total cellular RNA was isolated using the Qiagen RNeasy Mini Kit (Qiagen, Basel, Switzerland), which included a DNase digest. Total RNA was quantified spectrophotometrically and equal amounts (5 µg) were transcribed into cDNA with oligo(d)T primers and StrataScript RT Reverse Transcriptase using the StrataScript First-Strand Synthesis System (Stratagene, Rotkreuz, Switzerland). Duplicates of cDNA were amplified by RT-PCR with gene-specific primers using the 7500 Fast Real-Time PCR system (Applied Biosystems Inc., Rotkreuz, Switzerland) and the Power SYBR Green Master Mix (Applied Biosystems). Sequence-specific primers were selected using Primer Express v2.0 software (Applied Biosystems). The following primers were employed for glyceraldehyde-3-phosphate dehydrogenase (GAPDH): GAPDH forward, 5′-AAC AGC GAC ACC CAC TCC TC-3′; GAPDH reverse, 5′-GGA GGG GAG ATT CAG TGT GGT-3′. Primers for cytokines were as follows: IL1β-forward, 5′-CAG AAA ACA TGC CCG T-3′, IL1β reverse, 5′GCA CTA CCC TAA GGC AG-3′, IL8 forward, 5′-AGA CAG CAG AGC ACA CAA GC-3, IL8 reverse, 5′-ATG GTT CCT TCC GGT GGT-3′, MCP-1 forward, 5′-CCC CAG TCA CCT GCT GTT AT-3′, MCP-1 reverse, 5′-AGA TCT CCT TGG CCA CAA TG-3′, MIP1b forward, 5′-GAA AAC CTC TTT GCC ACC AA-3′, MIP1b reverse, 5′-TCA CTG GGA TCA GCA CAG AC-3′, HOXC10 forward, 5′-GAC CTG TGG TTC GTG C-3′, HOXC10 reverse, 5′-GCG GAT GGA TTC GAT CT-3′, TIGD4 forward, 5′-AGC AAC GAA GAG TGG T-3′, TIGD4 reverse, 5′-CTC TGG GGT TAC AGC C-3′, PCAF forward, 5′-GCA AGT CAA GGG CTA TGG-3′, PCAF reverse, 5′-GTG TAC GGG ATC CGT G-3′, CHMP4B forward, 5′-ACT TGT ACG GTA CTG GC-3′, CHMP4B reverse, 5′-TCG AGA TAT TTA ATA GAC AGT GC-3′, SOCS7 forward, 5′-CCG AAA GTT CTA CTA CTA TGA T-3′and SOCS7 reverse, 5′-AGA GTA CGG TCA TGT GC-3′. PCR was carried out with an initial denaturation step (10 min, 95°C) followed by 40 cycles of denaturation (15 sec, 95°C), annealing (30 sec, 55°C), and extension (30 sec, 72°C). Fluorescence was measured at the end of each extension. Relative mRNA levels were quantified by RQ Study SDS Software v1.3.1 (Applied Biosystems) using the comparative Ct method. The expression level of each gene was normalized to GAPDH levels in each experimental sample. Final data were expressed as mRNA expression in treated cells relative to expression in untreated cells. A melting curve analysis was performed for each amplicon to verify the specificity of each amplification step.

### Western blotting

For assaying HOXC10 and SOCS7I protein expression, cells were lysed with Mammalian Cell Lysis/Extraction reagent (Sigma-Aldrich Chemical Co., Buchs, Switzerland) supplemented with complete mini protease inhibitor cocktail tablets (Roche Diagnostics Schweiz AG, Rotkreuz, Switzerland). After clearing the lysates by high-speed centrifugation, protein concentrations of each sample were determined using a Protein Bradford assay (Bio-Rad Laboratories AG, Reinach, Switzerland). For immunoblotting, 20 µg total protein of each sample was resolved on SDS 4–15% gradient polyacrylamide gels and transferred to PVDF membrane (Millipore AG, Zug, Switzerland). After transfer, the membranes were incubated for 1 hr in blocking solution (5% non-fat milk in PBS containing 0.1% Tween-20) and then 1 hr with mouse anti-HOXC10 (1∶1000. abnova) or rabbit anti-SOCS7 (1∶1000, abcam) in blocking solution. Antibody binding was detected with a horseradish peroxidase-coupled sheep-anti rabbit or donkey-anti rabbit secondary antibody diluted 1∶10,000 followed by enhanced chemiluminescence (ECL) detection (ECL Plus, Amersham Pharmacia Biotech, Inc., Uppsala, Sweden).

### Measurement of cytokine production

The inflammatory cytokines IL-8, IL-1β, MIP-1β and MCP-1 were measured in undiluted monocyte supernatants using a multiplex assay on the BioPlex 2200 platform (Bio-Rad, Hercules, CA, USA), with commercial antibody-coated beads, standards, and reagents, according to the manufacturer's instructions (Bioplex human cytokine multiplex assay system, BioRad). Complete medium was used as blank. Data were analyzed on the Bioplex Reader using the BioPlex 3.0 software (Bio-Rad).

### Histone acetyltransferase (HAT) activity

HAT activity of nuclear extracts was measured with a colorimetric assay that measures NADH produced upon acetylation of a peptide substrate, following manufacturer recommendations (HAT Activity Colorimetric Assay Kit, BioVision, California, USA).

### Histone deacetylase (HDAC) activity

HDAC activity of nuclear extracts was measured with a colorimetric assay, following manufacturer recommendations (Colorimetric HDAC Activity Assay Kit, BioVision, California, USA).

### Statistical analysis

Data were analyzed with the use of Graphpad-Prism statistical software (version 4.0). We used an unpaired 2-tailed Student *t* ^-^test or, for comparison of data among groups, 1-way ANOVA followed by the Newman–Keuls test. Probability values <0.05 were considered statistically significant.

For array data, fold changes (up or down regulated) of gene expression were calculated using the re-ratio function of Rosetta Biosoftware that allows direct comparison between two samples (LPS/INFγ and LPS/INFγ/APC) that were both hybridized against a common reference (untreated control). All sequences regulated by LPS/INFγ/APC with a calculated fold change of more than 2.0 (p<0.01) were subjected to one-way ANOVA factorial analysis, compared with the significantly regulated sequences (fold change >2, *p* value<0.01) by LPS/INFγ.

## Results

### Differential gene expression of inflamed human macrophages

Human macrophages prepared from buffy coats of healthy blood donors were cultured for 24 hr and then stimulated with INF-γ and LPS. Unstimulated macrophages were used as the baseline control. The transcriptional profiles were then determined by microarray analysis using Agilent human 44k 60-mer oligonucleotide microarray chips. Profiles of identical experimental settings from three independent donors were analyzed with Rosetta biosoftware. After 8hr stimulation, these macrophages showed more than a 2-fold (p<0.01) change in expression in 2979 genes. Of the 2547 genes affected after 8 hr by INF/LPSγ stimulation, 1692 were up-regulated, (with a maximal change of 222.5-fold) and 1287 were down-regulated (with a maximal change of 18.0-fold). The maximally regulated genes were ankyrin repeat domain 22 (ANKRD22, 222-fold increased expression) and selenoprotein P (SEPP1, 18-fold decreased expression). The majority of differentially expressed genes were identified as unique and named in GenBank, whereas the remaining transcripts were either identified as unnamed expressed sequence tags or were hypothetical.

**Figure 1:**
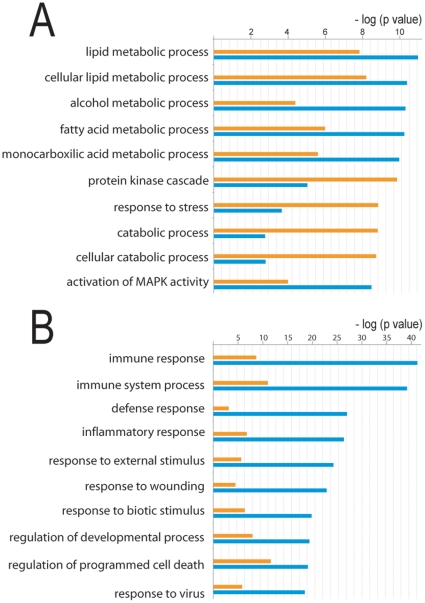
Functional clustering of genes regulated by LPS/INFγ. The gene expression profile of macrophages treated with LPS/INFγ compared with untreated control was analyzed with Rosetta Biosoftware [12]. Genes showing at least a 2.0 fold change were selected and characterized according to their GO biological process in the GeneGO software and compared with the classification of all genes present in the array in order to find which functional categories were significantly (p<0.05) over- or underrepresented [13] in the genes downregulated (**A**) or upregulated (**B**) by LPS/INFγ. The blue bars represent the genes regulated by LPS/INFγ and the orange bars represent all genes represented in the microarray.

To determine which functional categories are over-represented among regulated genes with a given statistical significance, we performed automated unbiased clustering using GeneGO software. Genes displaying at least 2.0-fold differential expression levels were classified into various categories based on the biological function(s) of the encoded protein to determine the global direction of the molecular response to inflammation (Figure 1). Classification according to GO biological processes revealed that most genes up-regulated by LPS/INFγ fall into the functional categories related to immune response and inflammatory response. In the down-regulated genes, there was an over-representation of the protein kinase cascade and response to stress functional categories as well as an under-representation of categories related to metabolic processes.

### Effect of activated protein C on inflamed macrophages

APC has been shown to down-regulate TNF production by macrophages and to inhibit the production of inflammatory cytokines and chemokines in LPS-stimulated THP-1 cells [14], [15].

**Figure 2:**
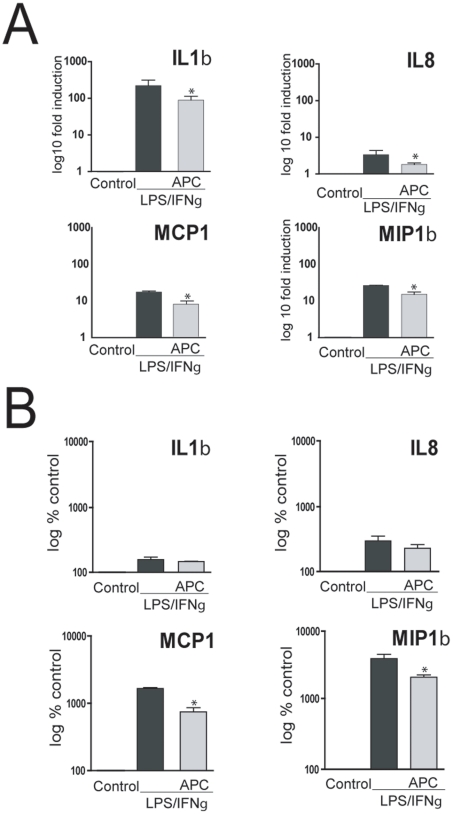
Regulation of inflammatory mediators by APC. (**A**) Fold induction of IL1β, IL8, MCP1 and MIP1 β mRNA expression in LPS/INFγ stimulated macrophages treated with and without APC for 8 hr. Changes in mRNA expression were normalized to changes in GAPDH expression and are expressed as mean ± SD from at least three independent experiments. *p<0.05 (**B**) Concentrations of IL1β, IL8, MCP1 and MIP1 β secreted by macrophages. Cells were stimulated with LPS/INFγ in the absence or presence of APC. Control cells were untreated. Cytokines were measured in cell culture supernatants collected 12 hr after treatment using the Bio-Plex Human Cytokine Multiplex Assay on the Bio-Plex 2200 platform. Cytokine concentrations in treated cells were normalized to the concentrations in control cells and are presented as mean ± SEM from three independent experiments. *p<0.05.

To examine the effect of APC in our experimental setup, real-time PCR was used to verify the mRNA levels of IL-1β and IL-8 as well as MCP-1 and MIP-1β in all samples used for microarray experiments. As shown in Figure 2, APC down-regulates the mRNA level of IL-1β, IL-8, MCP-1, and MIP-1β (Figure 2A) and the protein expression of MCP-1, and MIP-1β (Figure 2B) in macrophages stimulated with LPS/INFγ.

In order to define unknown targets of APC on inflamed macrophages, we compared the mRNA expression profiles of macrophages incubated with LPS/INFγ with or without APC. Re-ratio analysis using Rosetta Biosoftware followed by one-way ANOVA [12], revealed 570 genes differentially expressed by inflammatory activation and these were significantly (more than 2-fold change, p<0.05) up- or down-regulated by APC. Of the 570 genes affected by APC upon LPS/INFγ stimulation, 440 genes were up-regulated (with a maximal change of 14.8-fold), and 130 genes were down-regulated (with a maximal change of 26.4-fold). The maximally regulated genes were tigger transposable element derived 4 (TIGD4, 14.8-fold increased expression) and angiomotin like 1 (AMOTL1, 26.4-fold decreased expression). A complete list of the genes specifically regulated by APC is given as supplemental material (Table S1).

Among the genes down-regulated by APC is Wnt5A, a gene that recently emerged to be an important factor for the Toll-mediated inflammatory signaling in macrophages. A detailed study of the anti-inflammatory impact of Wnt5A regulation by APC has recently been described [12].

**Figure 3:**
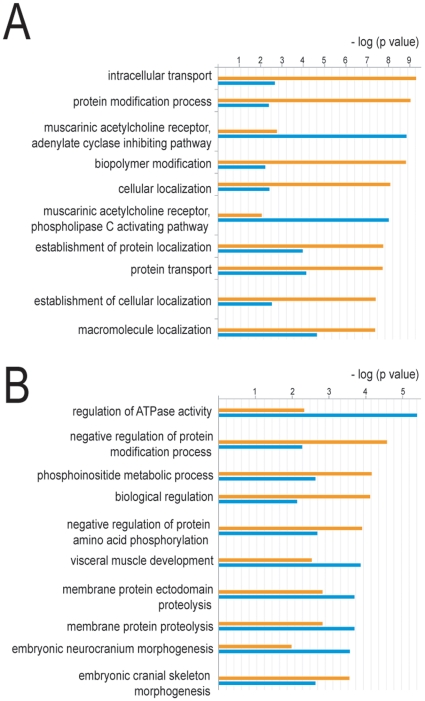
Functional clustering of genes regulated by APC in inflamed macrophages . The gene expression profile of macrophages treated with LPS/INFγ vs LPS/INFγ/APC was analyzed with Rosetta Biosoftware. Genes showing at least a 2.0 fold change were selected and characterized according to their GO biological process in the GeneGO software and compared with the classification of all genes present in the array in order to find which functional categories were significantly (p<0.05) over or under-represented in the genes downregulated (**A**) or upregulated (**B**) by APC. The blue bars represent the genes regulated by LPS/INFγ and the orange bars represent all genes present in the array.

To determine which functional clusters were over-represented among APC regulated genes, we applied the same analysis (GeneGO software) as in the genes regulated by LPS/INFγ (Figure 3). Among the genes down-regulated by APC, there was significant under-representation of genes involved in intracellular transport and protein transport and over-representation of genes involved in the adenylate inhibiting pathway and the phospholipase C activating pathway. The genes up-regulated by APC showed an over-representation of genes involved the regulation of ATPase activity and an under-representation of genes involved in phosphoinositide metabolic process and negative regulation of protein amino acid phosphorylation.

### Confirmation of microarray results by RT-PCR and Western Blot

**Figure 4:**
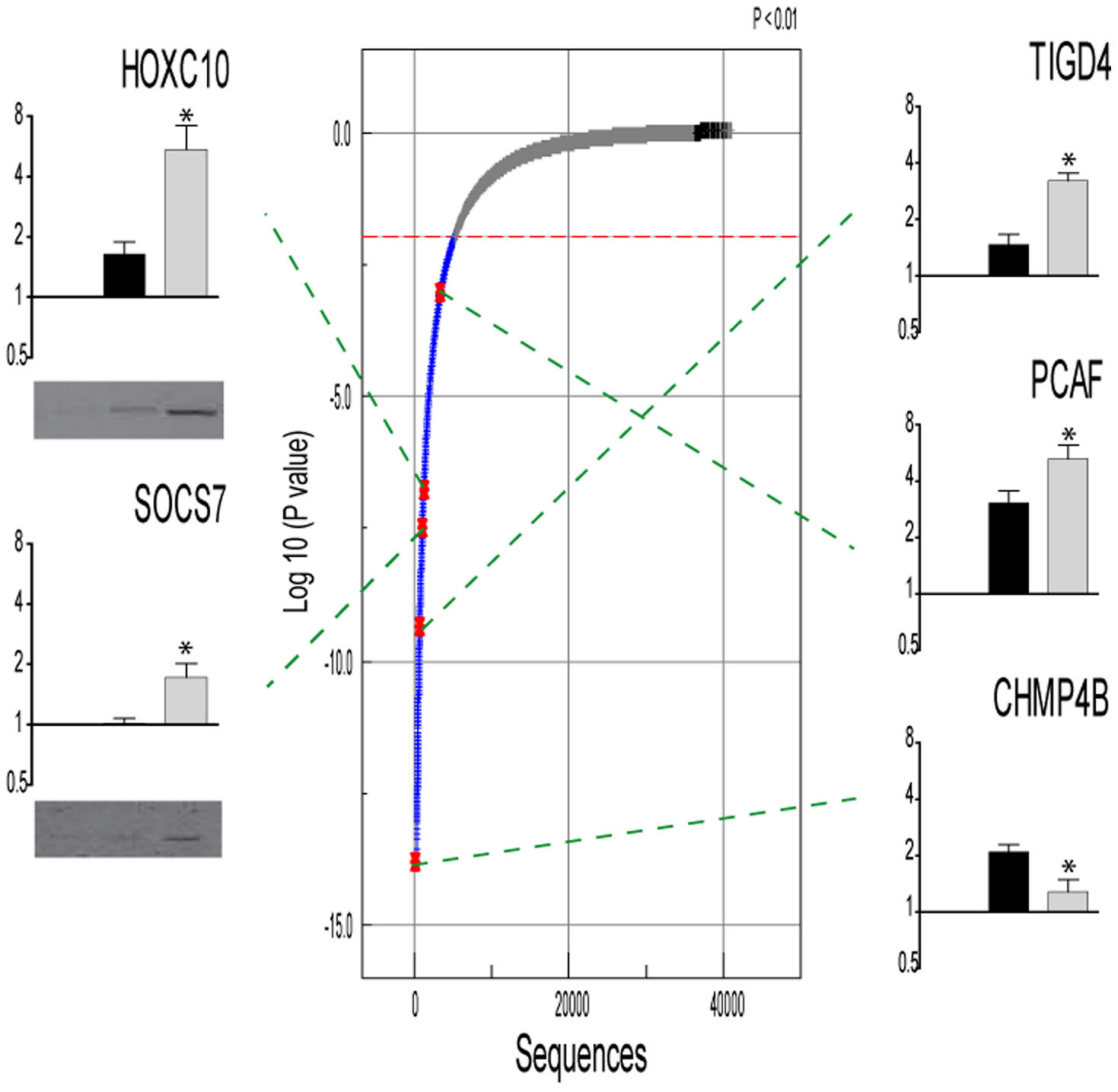
Statistical analysis of genes exclusively affected by APC in inflamed macrophages. In a first step of data analysis, fold changes (up or down regulated) of gene expression were calculated using the re-ratio function of Rosetta Biosoftware that allows direct comparison between two samples (LPS/INFγ and LPS/INFγ/APC) that were both hybridized against a common reference (untreated control). All sequences with a calculated fold change of more than 2.0 (p<0.01) were subjected to one-way ANOVA factorial analysis, compared with the significantly regulated sequences (fold change >2, *p* value<0.01) by LPS/INFγ. The ANOVA plot displays each gene (dot) in relation to its *p*-value (y axis) and the ranked sequence number (x-axis). Effects are considered significant at a *p*-value <0.01. Significantly regulated genes are plotted below the dashed red line. Highlighted genes, represented in red, were confirmed by real-time PCR (bar graphs) and when possible (antibody availability) by Western Blot.

Using quantitative real-time RT-PCR we confirmed the expression patterns obtained by microarray analysis for selected genes related to regulation of gene transcription (TIGD4, HOXC10, CHMP4B and PCAF) and cytokine secretion (SOCS7). The cytokine IL-10, which has well described anti-inflammatory properties, was used to compare its effect with the suspected but not elucidated anti-inflammatory action of APC in our setting of inflammatory activated macrophages. None of the selected genes were significantly regulated by IL10 or have been previously linked to the anti-inflammatory action of APC (Figure 4).

The regulation was also confirmed at the protein level (HOXC10 and SOCS7). For the other gene transcripts (TIGD4, CHMP4B and PCAF) no antibodies were available. APC significantly up-regulates TIGD4, PCAF and SOCS7 and HOXC10 and CHMP4B were significantly down-regulated by APC.

### APC alters the balance between acetylase and deacetylase activity in inflamed macrophages

Transcription of eukaryotic genes is complex and depends on different co-activators, such as p300, PCAF and c-AMP response element-binding protein, as well as histone acetyltransferases (HATs) and histone deacetylases (HDACs). HATs are responsible mainly for destabilizing the chromatin structure to allow accessibility to different transcription factors. In contrast, HDACs serve as co-repressors of gene transcription by restoring the condensation of DNA in chromatin.

**Figure 5:**
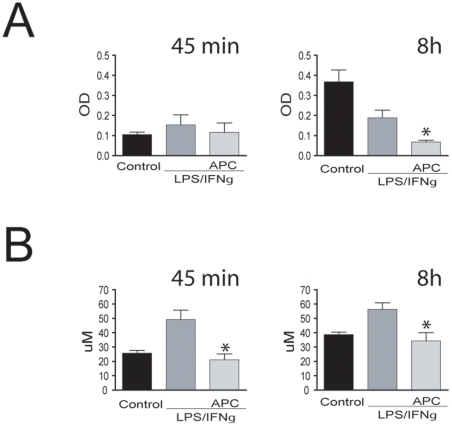
APC alters the histone acetyltransferase/deacetylase activity balance in inflamed macrophages. (A) Colorimetric determination of HAT activity in nuclear extracts of macrophages treated with LPS/INFγ with and without APC for 45 min (left) or 8 hr (right). Data are represented as mean ± SEM from three independent experiments. *p<0.05. (B) Colorimetric determination of HDAC activity in nuclear extracts of macrophages treated with LPS/INFγ with and without APC for 45 min (left) or 8 h (right). Data are represented as mean ± SEM from three independent experiments. *p<0.05.

Because PCAF was one of the genes significantly regulated by APC upon inflammatory stimulus and has acetyltransferase activity, we hypothesized that APC might regulate activity of HATs. As shown in Figure 5A, APC significantly downregulates histone acetyltransferase activity in nuclear extracts of treated macrophages after 8 hr but not after 45 min exposure. This indicates that APC has a long-term effect. As gene expression is regulated by a balance between HAT and HDACs, we also investigated APC effect on HDACs activity (Figure 5B) and found that APC inhibits HDAC activity in treated macrophages after 45 min and 8 hr stimulation suggesting a fast and prolonged effect of APC on HDAC activity.

## Discussion

Circulating macrophages play a major role in the immediate host response to invading microorganisms. The primary functions of macrophages include the phagocytosis of invading pathogens and the synthesis and secretion of pro-inflammatory cytokines, chemokines, and growth factors. In this study, we determined the transcriptional changes occurring in human macrophages after inflammatory stimulation (LPS and INFγ). The genes mostly regulated by inflammatory stimuli were ankyrin repeat domain 22 (increased expression) and selenoprotein P (decreased expression). The ankyrin-repeat domain (ARD) was first discovered as a repeated sequence in yeast cell-cycle regulation proteins [16], [17]. It is named after ankyrin, a cytoskeletal adapter protein, which contains 24 tandem copies of the repeat [17]. Since first being discovered, over 2800 ankyrin repeat proteins have been identified, each containing between three and 24 copies of the ankyrin repeat [18], [19]. Ankyrin repeats are common in signaling proteins, and appear to be general protein–protein interaction motifs [19]. The upregulation of ankyrin repeat domain 22 under inflammatory conditions is novel and may represent an increase of several proteins containing this domain that are involved in the inflammatory response, such as IκB [18]. Selenoprotein P is the major transporter of selenium, a trace element required for normal development [20]. The downregulation of selenoprotein P in our experimental setup is in line with previous findings that human and/or murine *Sepp1* gene promoter activity is impaired by IFN-γ, TNF-α, and IL-1β [21], [22] while induced by TGF-β [22] and IL-10 [23] suggesting a differential regulation of selenoprotein P expression during inflammatory reactions [18]. Functional clustering of genes significantly regulated by inflammatory stimuli, showed a significant over-representation of genes involved in the inflammatory response (upregulated genes) and genes associated with the lipid metabolic process (downregulated genes). The over-representation of downregulated genes associated with lipid metabolic processes highlights the fact that macrophages are an active source of pro- and anti-inflammatory lipid mediators. Modulation of genes involved in general cellular metabolic activities is a prominent feature of macrophage differentiation and polarization. Also, macrophages are a major component of adipose tissue and play a role in the metabolic syndrome [24].

We and others have focused on the effects of activated protein C (APC) on macrophages, cells that play a critical role in initiating, perpetuating, and modulating the immediate host response to invading microorganisms. Activated protein C has been shown to exert direct anti-inflammatory effects on macrophages by inhibiting the production of pro-inflammatory cytokines [25], [26]. Using a genome wide approach we tried to identify novel targets for the anti-inflammatory action of APC. The genes most highly regulated by APC were TIGD4 (increased) and angiomotin like 1 (decreased). TIGD4 belongs to the tigger subfamily of the pogo superfamily of DNA-mediated transposons in humans [27]. These proteins are related to DNA transposons found in fungi and nematodes. They are also very similar to the major mammalian centromere protein B [27]. Other novel APC targets were chromatin binding protein 4B (CHMP4B), and p300/CBP-associated factor (PCAF), which may indicate a role of APC in the epigenetic control of gene transcription. It is known that PCAF has histone acetyltransferase activity, and through an acetyltransferase activity assay we were able to show that APC inhibits histone acetyltransferase activity in nuclear extracts from inflamed macrophages (Figure 5A). In health, the expression of genes coding for many pro-inflammatory cytokines remains silent. Mechanisms of transcriptional repression dominate until overcome by stimulation from extracellular stress signals such as microbial products via the Toll like receptors. Although several transcription factors initiate *de novo* expression of pro-inflammatory cytokines, synthesis and secretion are tightly controlled events. Therefore, any mechanism for cytokine production is a potential target for acetylation and histone acetyltransferase (HAT) and deacetylase (HDAC) activity regulation. Acetylation of transcription factors can alter interactions between transcription factors and DNA and among different transcription factors, and is an integral part of the transcription and differentiation processes. HATs are responsible mainly for destabilizing the chromatin structure to allow accessibility of different transcription factors, including NF-kB to the transcriptional site in the DNA. NF-kB can induce histone acetylation and other histone modifications in a temporal manner [28] leading to recruitment of other co-activator and re-modeling complexes and the induction of inflammatory gene expression [29]. A recent study showed that HAT activity would be required to ensure the time-dependent induction of the inflammatory genes IL6, IL8 [30], [31], therefore an inhibition of HAT activity would contribute to the downregulation of these pro-inflammatory cytokines.

The inhibition of HDAC activity by APC was unexpected, but several experimental studies have demonstrated that HDAC inhibitors can modulate immune responses [32], [33], [34]. Recent studies have shown that pre-treatment with suberoylanilide hydroxamic acid (SAHA), an HDAC inhibitor, significantly reduced LPS-induced secretion of TNF-α by peritoneal macrophages [32] by impairing transcription factor recruitment [35]. Our results suggest that APC exerts its anti-inflammatory action by altering the acetylation balance in inflamed macrophages.

Among the genes regulated by APC was also the homeobox domain C10 (HOXC10). HOXC10 is one of the highly conserved HOXC family members of transcription factors that play an important role in morphogenesis, cell differentiation, and proliferation [36], [37], [38]. The HOXC protein levels are controlled during cell differentiation and proliferation. Selected Hox proteins have been shown to directly and indirectly regulate the expression of many angiogenic and growth factors, including basic fibroblast growth factor, vascular endothelial growth factor, IL8, and Ang2 [38]. The functional role of HOXC10 in inflammation is a question that needs to be clarified in further studies.

SOCS (suppressor of cytokine signaling) proteins are known to act as negative regulators of cytokine action via inhibition of JAK/STAT signaling [39]. The SOCS family consists of 8 proteins (cytokine-inducible SH2-containing protein [CIS] and SOCS1-SOCS7). There is very little known about the function of SOCS7. It has been shown to interact with vinexin [40]. As vinexin is involved in the signal transduction from EGF-R to JNK and in cytoskeletal organization and cell spreading, SOCS7 could modulate these functions and thus play a role in adhesion-dependent signaling and in cytoskeletal remodeling in normal and transformed cells [41]. The observation that SOCS7 expression is induced by APC is novel and precise consequences resulting from this induction should be further elucidated.

## Supporting Information

Table S1 Genes significantly (p<0.01) regulated by APC in inflamed macrophages (DOC)
