# Supplementary material for: Transcriptome Analysis Revealed Unique Genes as Targets for the Anti-inflammatory Action of Activated Protein C in Human Macrophages
Source: PLoS One. 2010 Oct 15;5(10):e15352. doi: 10.1371/journal.pone.0015352 (PMC2955553; doi:10.1371/journal.pone.0015352)
Supplement: Table S1 — Genes significantly (p<0.01) regulated by APC in inflamed macrophages (DOC) [file pone.0015352.s001.doc]

**Supplemental Data**

**Table I**

Genes significantly (p<0.01) regulated by APC in inflamed macrophages

| **Sequence name** | **Accession #** | **Sequence description** | **Fold Change** |
| --- | --- | --- | --- |
| AMOTL1 | NM_130847 | Homo sapiens angiomotin like 1 | -26.43 |
| NRXN1 | NM_004801 | Homo sapiens neurexin 1 | -10.09 |
| ANKRD22 | NM_144590 | Homo sapiens ankyrin repeat domain 22 | -9.69 |
| MCF2L2 | NM_015078 | Homo sapiens MCF.2 cell line derived transforming sequence-like 2 | -7.09 |
| MBD3L2 | NM_144614 | Homo sapiens methyl-CpG binding domain protein 3-like 2 | -7.09 |
| ATAD2 | NM_014109 | Homo sapiens ATPase family, AAA domain containing 2 | -5.78 |
| NUP160 | NM_015231 | Homo sapiens nucleoporin 160kDa | -4.85 |
| GBP4 | NM_052941 | Homo sapiens guanylate binding protein 4 | -4.54 |
| AGT | NM_000029 | Homo sapiens angiotensinogen (serine (or cysteine) proteinase inhibitor | -4.36 |
| FST | NM_013409 | Homo sapiens follistatin | -4.25 |
| SSBP1 | BC008402 | Homo sapiens single-stranded DNA binding protein 1 | -4.23 |
| PTGER3 | NM_000957 | Homo sapiens prostaglandin E receptor 3 (subtype EP3) | -4.22 |
| ROBO1 | NM_133631 | Homo sapiens roundabout, axon guidance receptor, homolog 1 (Drosophila) | -4.00 |
| ARHGEF7 | NM_145735 | Homo sapiens Rho guanine nucleotide exchange factor (GEF) 7 | -3.98 |
| SLC13A1 | NM_022444 | Homo sapiens solute carrier family 13 (sodium/sulfate symporters), member 1 | -3.70 |
| ITGA1 | NM_181501 | Homo sapiens integrin, alpha 1 | -3.68 |
| TEKT3 | NM_031898 | Homo sapiens tektin 3 | -3.56 |
| HIF3A | NM_152794 | Homo sapiens hypoxia inducible factor 3, alpha subunit | -3.50 |
| VSNL1 | NM_003385 | Homo sapiens visinin-like 1 | -3.41 |
| JMJD2D | NM_018039 | Homo sapiens jumonji domain containing 2D | -3.37 |
| IL12A | NM_000882 | Homo sapiens interleukin 12A | -3.31 |
| HMGA1 | NM_002131 | Homo sapiens high mobility group AT-hook 1 | -3.30 |
| IL12B | NM_002187 | Homo sapiens interleukin 12B | -3.29 |
| CCL13 | NM_005408 | Homo sapiens chemokine (C-C motif) ligand 13 | -3.15 |
| NRG2 | NM_013982 | Homo sapiens neuregulin 2 | -2.97 |
| PLS3 | NM_005032 | Homo sapiens plastin 3 (T isoform) | -2.85 |
| SH3GL2 | NM_003026 | Homo sapiens SH3-domain GRB2-like 2 | -2.82 |
| RBM11 | NM_144770 | Homo sapiens RNA binding motif protein 11 | -2.82 |
| ZNF326 | NM_182975 | Homo sapiens zinc finger protein 326 | -2.75 |
| RNF138 | NM_016271 | Homo sapiens ring finger protein 138 | -2.75 |
| NXF5 | NM_032946 | Homo sapiens nuclear RNA export factor 5 | -2.75 |
| SPANXA1 | NM_013453 | Homo sapiens sperm protein associated with the nucleus, X-linked, family member A1 | -2.75 |
| GPR88 | NM_022049 | Homo sapiens G-protein coupled receptor 88 | -2.74 |
| SSX2 | NM_175698 | Homo sapiens synovial sarcoma, X breakpoint 2 | -2.74 |
| ABCC4 | NM_005845 | Homo sapiens ATP-binding cassette, sub-family C (CFTR/MRP), member 4 | -2.71 |
| WNT5A | NM_003392 | Homo sapiens wingless-type MMTV integration site family, member 5A | -2.71 |
| STK32A | NM_145001 | Homo sapiens serine/threonine kinase 32A | -2.69 |
| BF | NM_001710 | Homo sapiens B-factor, properdin | -2.66 |
| IMPA1 | NM_005536 | Homo sapiens inositol(myo)-1(or 4)-monophosphatase 1 | -2.65 |
| TARP | NM_001003799 | Homo sapiens TCR gamma alternate reading frame protein | -2.59 |
| AD031 | NM_032021 | Homo sapiens AD031 protein | -2.57 |
| ZRANB3 | NM_032143 | Homo sapiens zinc finger, RAN-binding domain containing 3 | -2.54 |
| CYP3A4 | NM_017460 | Homo sapiens cytochrome P450, family 3, subfamily A, polypeptide 4 | -2.54 |
| PTPN3 | NM_002829 | Homo sapiens protein tyrosine phosphatase, non-receptor type 3 | -2.53 |
| ETV7 | NM_016135 | Homo sapiens ets variant gene 7 | -2.53 |
| GPSM2 | NM_013296 | Homo sapiens G-protein signalling modulator 2 (AGS3-like, C. elegans) | -2.51 |
| CSNK2A1 | NM_177559 | Homo sapiens casein kinase 2, alpha 1 polypeptide | -2.51 |
| TRIP13 | NM_004237 | Homo sapiens thyroid hormone receptor interactor 13 | -2.51 |
| SPAG1 | NM_003114 | Homo sapiens sperm associated antigen 1 | -2.50 |
| ALPK2 | NM_052947 | Homo sapiens heart alpha-kinase | -2.48 |
| SAMD3 | NM_152552 | Homo sapiens sterile alpha motif domain containing 3 | -2.45 |
| SIX4 | NM_017420 | Homo sapiens sine oculis homeobox homolog 4 | -2.43 |
| CHMP4B | NM_176812 | Homo sapiens chromatin modifying protein 4B | -2.42 |
| ATP2B3 | NM_021949 | Homo sapiens ATPase, Ca++ transporting, plasma membrane 3 | -2.42 |
| NR1I2 | NM_003889 | Homo sapiens nuclear receptor subfamily 1, group I, member 2 | -2.41 |
| ZNF215 | NM_013250 | Homo sapiens zinc finger protein 215 | -2.41 |
| EAF1 | NM_033083 | Homo sapiens ELL associated factor 1 | -2.38 |
| CHRNA3 | NM_000743 | Homo sapiens cholinergic receptor, nicotinic, alpha polypeptide 3 | -2.34 |
| GMFB | NM_004124 | Homo sapiens glia maturation factor, beta | -2.33 |
| CXCL9 | NM_002416 | Homo sapiens chemokine (C-X-C motif) ligand 9 | -2.32 |
| CALD1 | NM_033138 | Homo sapiens caldesmon 1 | -2.32 |
| GULP1 | NM_016315 | Homo sapiens GULP, engulfment adaptor PTB domain containing 1 | -2.32 |
| TMEFF1 | NM_003692 | Homo sapiens transmembrane protein with EGF-like and two follistatin-like domains 1 | -2.31 |
| GRIK2 | NM_175768 | Homo sapiens glutamate receptor, ionotropic, kainate 2 | -2.31 |
| TCF7L1 | NM_031283 | Homo sapiens transcription factor 7-like 1 (T-cell specific, HMG-box) | -2.29 |
| GNPNAT1 | NM_198066 | Homo sapiens glucosamine-phosphate N-acetyltransferase 1 | -2.29 |
| MAGEA9 | NM_005365 | Homo sapiens melanoma antigen family A, 9 | -2.26 |
| LHX2 | NM_004789 | Homo sapiens LIM homeobox 2 | -2.24 |
| IFNG | NM_000619 | Homo sapiens interferon, gamma | -2.23 |
| PLVAP | NM_031310 | Homo sapiens plasmalemma vesicle associated protein | -2.23 |
| TECTA | NM_005422 | Homo sapiens tectorin alpha | -2.23 |
| IGFBP4 | NM_001552 | Homo sapiens insulin-like growth factor binding protein 4 | -2.22 |
| SYT4 | NM_020783 | Homo sapiens synaptotagmin IV | -2.22 |
| PRL | NM_000948 | Homo sapiens prolactin | -2.21 |
| PAK7 | NM_020341 | Homo sapiens p21(CDKN1A)-activated kinase 7 | -2.20 |
| CHRM2 | NM_001006630 | Homo sapiens cholinergic receptor, muscarinic 2 | -2.20 |
| FABP4 | NM_001442 | Homo sapiens fatty acid binding protein 4, adipocyte | -2.19 |
| CCNG1 | NM_004060 | Homo sapiens cyclin G1 | -2.19 |
| SUCNR1 | NM_033050 | Homo sapiens succinate receptor 1 | -2.19 |
| PIP5K1B | NM_003558 | Homo sapiens phosphatidylinositol-4-phosphate 5-kinase, type I, beta | -2.19 |
| SCGB1D1 | NM_006552 | Homo sapiens secretoglobin, family 1D, member 1 | -2.18 |
| ZD52F10 | NM_033317 | Homo sapiens dermokine | -2.18 |
| MRPL1 | NM_020236 | Homo sapiens mitochondrial ribosomal protein L1 | -2.17 |
| PBEF1 | NM_005746 | Homo sapiens pre-B-cell colony enhancing factor 1 | -2.17 |
| PCDH11Y | NM_032973 | Homo sapiens protocadherin 11 Y-linked | -2.17 |
| BARHL2 | NM_020063 | Homo sapiens BarH-like 2 (Drosophila) | -2.16 |
| ANKRD17 | NM_032217 | Homo sapiens ankyrin repeat domain 17 | -2.16 |
| TM4SF10 | NM_031442 | Homo sapiens transmembrane protein 47 | -2.15 |
| GDNF | NM_000514 | Homo sapiens glial cell derived neurotrophic factor | -2.15 |
| LDHAL6A | NM_144972 | Homo sapiens lactate dehydrogenase A-like 6A | -2.15 |
| KRT6L | NM_175834 | Homo sapiens keratin 6L | -2.15 |
| MAP3K13 | NM_004721 | Homo sapiens mitogen-activated protein kinase kinase kinase 13 | -2.12 |
| CDS1 | NM_001263 | Homo sapiens CDP-diacylglycerol synthase (phosphatidate cytidylyltransferase) 1 | -2.12 |
| EIF3S6 | NM_001568 | Homo sapiens eukaryotic translation initiation factor 3, subunit 6 48kDa | -2.12 |
| PCTK2 | NM_002595 | Homo sapiens PCTAIRE protein kinase 2 | -2.12 |
| SERP1 | NM_014445 | Homo sapiens stress-associated endoplasmic reticulum protein 1 | -2.11 |
| BMPR2 | NM_001204 | Homo sapiens bone morphogenetic protein receptor, type II (serine/threonine kinase) | -2.11 |
| RKHD2 | NM_016626 | Homo sapiens ring finger and KH domain containing 2 | -2.11 |
| GFRA2 | NM_001495 | Homo sapiens GDNF family receptor alpha 2 | -2.10 |
| ATP6V1C2 | NM_144583 | Homo sapiens ATPase, H+ transporting, lysosomal 42kDa, V1 subunit C isoform 2 | -2.10 |
| HSD17B12 | NM_016142 | Homo sapiens hydroxysteroid (17-beta) dehydrogenase 12 | -2.09 |
| NURIT | NM_152719 | Homo sapiens testis-specific leucine zipper protein nurit | -2.09 |
| INPP5A | NM_005539 | Homo sapiens inositol polyphosphate-5-phosphatase, 40kDa | -2.09 |
| TNFAIP9 | NM_024636 | Homo sapiens tumor necrosis factor, alpha-induced protein 9 | -2.08 |
| IL1A | NM_000575 | Homo sapiens interleukin 1, alpha | -2.08 |
| TWSG1 | NM_020648 | Homo sapiens twisted gastrulation homolog 1 | -2.08 |
| RNF144 | NM_014746 | Homo sapiens ring finger protein 144 | -2.08 |
| DDI1 | NM_001001711 | Homo sapiens DNA-damage inducible protein 1 | -2.08 |
| IMPA1 | NM_005536 | Homo sapiens inositol(myo)-1(or 4)-monophosphatase 1 | -2.07 |
| NDST1 | NM_001543 | Homo sapiens N-deacetylase/N-sulfotransferase (heparan glucosaminyl) 1 | -2.07 |
| SORBS1 | NM_006434 | Homo sapiens sorbin and SH3 domain containing 1 | -2.07 |
| CLK4 | NM_020666 | Homo sapiens CDC-like kinase 4 | -2.07 |
| CCNC | NM_005190 | Homo sapiens cyclin C | -2.06 |
| DACH1 | NM_080759 | Homo sapiens dachshund homolog 1 (Drosophila) | -2.06 |
| UBQLN1 | NM_013438 | Homo sapiens ubiquilin 1 | -2.05 |
| CSF2 | NM_000758 | Homo sapiens colony stimulating factor 2 (granulocyte-macrophage) | -2.05 |
| HLA-DOA | NM_002119 | Homo sapiens major histocompatibility complex, class II, DO alpha | -2.05 |
| TSNAX | NM_005999 | Homo sapiens translin-associated factor X | -2.05 |
| CYCS | NM_018947 | Homo sapiens cytochrome c, somatic (CYCS), nuclear gene encoding mitochondrial protein | -2.05 |
| COL15A1 | NM_001855 | Homo sapiens collagen, type XV, alpha 1 | -2.04 |
| PITPNM1 | NM_004910 | Homo sapiens phosphatidylinositol transfer protein, membrane-associated 1 | -2.04 |
| MCP | NM_172350 | Homo sapiens membrane cofactor protein (CD46, trophoblast-lymphocyte cross-reactive antigen) (MCP) | -2.03 |
| LMO3 | NM_018640 | Homo sapiens LIM domain only 3 (rhombotin-like 2) | -2.02 |
| RAB5A | NM_004162 | Homo sapiens RAB5A, member RAS oncogene family | -2.02 |
| DPCR1 | NM_080870 | Homo sapiens diffuse panbronchiolitis critical region 1 | -2.02 |
| AP4S1 | NM_007077 | Homo sapiens adaptor-related protein complex 4, sigma 1 subunit | -2.02 |
| SGCB | NM_000232 | Homo sapiens sarcoglycan, beta (43kDa dystrophin-associated glycoprotein) | -2.02 |
| TMEM22 | NM_025246 | Homo sapiens transmembrane protein 22 | -2.01 |
| RAB39 | NM_017516 | Homo sapiens RAB39, member RAS oncogene family | -2.01 |
| PLSCR4 | NM_020353 | Homo sapiens phospholipid scramblase 4 | -2.01 |
| ACMSD | NM_138326 | Homo sapiens aminocarboxymuconate semialdehyde decarboxylase | 2.00 |
| BAAT | NM_001701 | Homo sapiens bile acid Coenzyme A: amino acid N-acyltransferase (glycine N-choloyltransferase) | 2.00 |
| UBQLN4 | NM_020131 | Homo sapiens ubiquilin 4 | 2.00 |
| APRG1 | NM_178344 | Homo sapiens AP20 region protein | 2.01 |
| GREM2 | NM_022469 | Homo sapiens gremlin 2 homolog, cysteine knot superfamily (Xenopus laevis) | 2.01 |
| ST6GAL2 | NM_032528 | Homo sapiens ST6 beta-galactosamide alpha-2,6-sialyltranferase 2 | 2.01 |
| ZNF174 | NM_003450 | Homo sapiens zinc finger protein 174 | 2.01 |
| MAGEC3 | NM_138702 | Homo sapiens melanoma antigen, family C, 3 | 2.01 |
| TCF8 | NM_030751 | Homo sapiens transcription factor 8 (represses interleukin 2 expression) | 2.01 |
| NANOG | NM_024865 | Homo sapiens Nanog homeobox | 2.02 |
| SULT2A1 | NM_003167 | Homo sapiens sulfotransferase family, cytosolic, 2A, dehydroepiandrosterone (DHEA)-preferring, member 1 | 2.02 |
| ATP13A4 | NM_032279 | Homo sapiens ATPase type 13A4 | 2.02 |
| PTPN14 | NM_005401 | Homo sapiens protein tyrosine phosphatase, non-receptor type 14 | 2.02 |
| BTNL8 | NM_024850 | Homo sapiens butyrophilin-like 8 (BTNL8), mRNA [NM_024850] | 2.02 |
| KCNH8 | NM_144633 | Homo sapiens potassium voltage-gated channel, subfamily H (eag-related), member 8 | 2.02 |
| BVES | NM_147147 | Homo sapiens blood vessel epicardial substance | 2.02 |
| SST | NM_001048 | Homo sapiens somatostatin | 2.02 |
| NID2 | NM_007361 | Homo sapiens nidogen 2 (osteonidogen) | 2.02 |
| TEAD4 | NM_003213 | Homo sapiens TEA domain family member 4 | 2.02 |
| PGBD4 | NM_152595 | Homo sapiens piggyBac transposable element derived 4 | 2.02 |
| APH1A | NM_016022 | Homo sapiens anterior pharynx defective 1 homolog A (C. elegans) | 2.02 |
| PCK1 | NM_002591 | Homo sapiens phosphoenolpyruvate carboxykinase 1 (soluble) | 2.02 |
| TFAP2A | NM_003220 | Homo sapiens transcription factor AP-2 alpha (activating enhancer binding protein 2 alpha) | 2.02 |
| MAGEB4 | NM_002367 | Homo sapiens melanoma antigen family B, 4 | 2.02 |
| TEKT1 | NM_053285 | Homo sapiens tektin 1 | 2.03 |
| IGSF2 | NM_004258 | Homo sapiens immunoglobulin superfamily, member 2 | 2.03 |
| TGM5 | NM_201631 | Homo sapiens transglutaminase 5 | 2.03 |
| LAX | NM_017773 | Homo sapiens lymphocyte transmembrane adaptor 1 | 2.03 |
| CDH17 | NM_004063 | Homo sapiens cadherin 17, LI cadherin (liver-intestine) | 2.03 |
| RS1 | NM_000330 | Homo sapiens retinoschisis (X-linked, juvenile) 1 | 2.03 |
| NHLH1 | NM_005598 | Homo sapiens nescient helix loop helix 1 | 2.03 |
| NEDD4 | NM_006154 | Homo sapiens neural precursor cell expressed, developmentally down-regulated 4 | 2.04 |
| TSCOT | NM_033051 | Homo sapiens thymic stromal co-transporter | 2.04 |
| PCAF | NM_003884 | Homo sapiens p300/CBP-associated factor | 2.04 |
| TAT | NM_000353 | Homo sapiens tyrosine aminotransferase | 2.05 |
| TNP1 | NM_003284 | Homo sapiens transition protein 1 (during histone to protamine replacement) | 2.05 |
| IL1RAP | NM_002182 | Homo sapiens interleukin 1 receptor accessory protein | 2.05 |
| PCDH9 | NM_020403 | Homo sapiens protocadherin 9 | 2.05 |
| NM_001015886 | NM_001015886 | Homo sapiens high mobility group AT-hook 2 | 2.05 |
| DEPDC1 | NM_017779 | Homo sapiens DEP domain containing 1 | 2.05 |
| RNF8 | NM_003958 | Homo sapiens ring finger protein 8 | 2.05 |
| SLC9A6 | NM_006359 | Homo sapiens solute carrier family 9 (sodium/hydrogen exchanger), isoform 6 | 2.05 |
| ZNF354C | NM_014594 | Homo sapiens zinc finger protein 354C | 2.05 |
| SLC9A3 | NM_004174 | Homo sapiens solute carrier family 9 (sodium/hydrogen exchanger), isoform 3 | 2.06 |
| MAP2K5 | NM_145161 | Homo sapiens mitogen-activated protein kinase kinase 5 | 2.06 |
| LGR5 | NM_003667 | Homo sapiens leucine-rich repeat-containing G protein-coupled receptor 5 | 2.06 |
| TNFSF18 | NM_005092 | Homo sapiens tumor necrosis factor (ligand) superfamily, member 18 | 2.06 |
| RNF133 | NM_139175 | Homo sapiens ring finger protein 133 | 2.06 |
| MYRIP | NM_015460 | Homo sapiens myosin VIIA and Rab interacting protein | 2.06 |
| NM_015111 | NM_015111 | Homo sapiens Nedd4 binding protein 3 | 2.06 |
| CKLFSF2 | NM_144673 | Homo sapiens chemokine-like factor super family 2 | 2.06 |
| HOXC12 | NM_173860 | Homo sapiens homeo box C12 | 2.07 |
| KCND3 | NM_004980 | Homo sapiens potassium voltage-gated channel, Shal-related subfamily, member 3 | 2.07 |
| LIPH | NM_139248 | Homo sapiens lipase, member H | 2.07 |
| PF4V1 | NM_002620 | Homo sapiens platelet factor 4 variant 1 | 2.07 |
| PTK2 | NM_153831 | Homo sapiens PTK2 protein tyrosine kinase 2 | 2.07 |
| FOXD4 | NM_207305 | Homo sapiens forkhead box D4 | 2.07 |
| CALML6 | NM_138705 | Homo sapiens calmodulin-like 6 | 2.08 |
| DGKH | NM_152910 | Homo sapiens diacylglycerol kinase, eta | 2.08 |
| CHRM2 | NM_001006627 | Homo sapiens cholinergic receptor, muscarinic 2 | 2.08 |
| GTPBP2 | NM_019096 | Homo sapiens GTP binding protein 2 | 2.08 |
| HS3ST5 | NM_153612 | Homo sapiens heparan sulfate (glucosamine) 3-O-sulfotransferase 5 | 2.08 |
| MYH7B | NM_020884 | Homo sapiens myosin, heavy polypeptide 7B, cardiac muscle, beta | 2.08 |
| DAO | NM_001917 | Homo sapiens D-amino-acid oxidase | 2.08 |
| FLJ22800 | NM_024795 | Homo sapiens transmembrane 4 L six family member 20 | 2.08 |
| PAX3 | NM_181458 | Homo sapiens paired box gene 3 (Waardenburg syndrome 1) | 2.09 |
| SAA1 | NM_000331 | Homo sapiens serum amyloid A1 | 2.09 |
| PITPNM3 | NM_031220 | Homo sapiens PITPNM family member 3 | 2.09 |
| WNK4 | NM_032387 | Homo sapiens WNK lysine deficient protein kinase 4 | 2.09 |
| CRSP2 | NM_004229 | Homo sapiens cofactor required for Sp1 transcriptional activation, subunit 2, 150kDa | 2.10 |
| SYCP2 | NM_014258 | Homo sapiens synaptonemal complex protein 2 | 2.10 |
| EDA2R | NM_021783 | Homo sapiens ectodysplasin A2 receptor | 2.10 |
| ACACB | NM_001093 | Homo sapiens acetyl-Coenzyme A carboxylase beta | 2.10 |
| SFXN2 | NM_178858 | Homo sapiens sideroflexin 2 | 2.10 |
| FBI4 | NM_205857 | Homo sapiens FBI4 protein | 2.10 |
| ABL2 | NM_007314 | Homo sapiens v-abl Abelson murine leukemia viral oncogene homolog 2 (arg, Abelson-related gene) | 2.10 |
| GABRB3 | NM_000814 | Homo sapiens gamma-aminobutyric acid (GABA) A receptor, beta 3 | 2.10 |
| LRRTM2 | NM_015564 | Homo sapiens leucine rich repeat transmembrane neuronal 2 | 2.11 |
| HPGD | NM_000860 | Homo sapiens hydroxyprostaglandin dehydrogenase 15-(NAD) | 2.11 |
| ATP1B4 | NM_012069 | Homo sapiens ATPase, (Na+)/K+ transporting, beta 4 polypeptide | 2.11 |
| IPF1 | NM_000209 | Homo sapiens insulin promoter factor 1, homeodomain transcription factor | 2.11 |
| RBM24 | NM_153020 | Homo sapiens RNA binding motif protein 24 | 2.11 |
| BICD1 | NM_001003398 | Homo sapiens bicaudal D homolog 1 | 2.11 |
| MYO5C | NM_018728 | Homo sapiens myosin VC | 2.11 |
| CASP12P1 | NR_000035 | Homo sapiens caspase 12 pseudogene 1 | 2.11 |
| RAB11FIP4 | NM_032932 | Homo sapiens RAB11 family interacting protein 4 (class II) | 2.11 |
| MSC | NM_005098 | Homo sapiens musculin (activated B-cell factor-1) | 2.11 |
| HSF2 | NM_004506 | Homo sapiens heat shock transcription factor 2 | 2.12 |
| PTPN14 | NM_005401 | Homo sapiens protein tyrosine phosphatase, non-receptor type 14 | 2.12 |
| ANGPT1 | NM_139290 | Homo sapiens angiopoietin 1 | 2.12 |
| TRIM54 | NM_187841 | Homo sapiens tripartite motif-containing 54 | 2.12 |
| TBX22 | NM_016954 | Homo sapiens T-box 22 | 2.13 |
| MLLT7 | NM_005938 | Homo sapiens myeloid/lymphoid or mixed-lineage leukemia (trithorax homolog, Drosophila); translocated to, 7 | 2.13 |
| SELE | NM_000450 | Homo sapiens selectin E (endothelial adhesion molecule 1) | 2.13 |
| TCF2 | NM_006481 | Homo sapiens transcription factor 2, hepatic; LF-B3; variant hepatic nuclear factor | 2.13 |
| CFHL2 | NM_005666 | Homo sapiens complement factor H-related 2 | 2.13 |
| ZNF233 | NM_181756 | Homo sapiens zinc finger protein 233 | 2.13 |
| SPINK4 | NM_014471 | Homo sapiens serine protease inhibitor, Kazal type 4 | 2.14 |
| CABP7 | NM_182527 | Homo sapiens calcium binding protein 7 | 2.14 |
| PLA2G12B | NM_032562 | Homo sapiens phospholipase A2, group XIIB | 2.14 |
| PRKAA2 | NM_006252 | Homo sapiens protein kinase, AMP-activated, alpha 2 catalytic subunit | 2.15 |
| SEC14L3 | NM_174975 | Homo sapiens SEC14-like 3 (S. cerevisiae) | 2.15 |
| EVE1 | NM_001009555 | Homo sapiens SH3 domain protein D19 | 2.16 |
| HOXC9 | NM_006897 | Homo sapiens homeo box C9 | 2.16 |
| SLC25A27 | NM_004277 | Homo sapiens solute carrier family 25, member 27 | 2.17 |
| GLRA2 | NM_002063 | Homo sapiens glycine receptor, alpha 2 | 2.17 |
| SPATA9 | NM_031952 | Homo sapiens spermatogenesis associated 9 | 2.17 |
| SYTL5 | NM_138780 | Homo sapiens synaptotagmin-like 5 | 2.18 |
| CLCN4 | NM_001830 | Homo sapiens chloride channel 4 | 2.18 |
| FLJ10052 | NM_017982 | Homo sapiens sushi domain containing 4 | 2.18 |
| ASPM | NM_018136 | Homo sapiens asp (abnormal spindle)-like, microcephaly associated (Drosophila) | 2.18 |
| DMP1 | NM_004407 | Homo sapiens dentin matrix acidic phosphoprotein | 2.18 |
| LRRC2 | NM_024750 | Homo sapiens leucine rich repeat containing 2 | 2.18 |
| DDX53 | NM_182699 | Homo sapiens DEAD (Asp-Glu-Ala-Asp) box polypeptide 53 | 2.19 |
| CEACAM7 | NM_006890 | Homo sapiens carcinoembryonic antigen-related cell adhesion molecule 7 | 2.19 |
| RBMS3 | NM_014483 | Homo sapiens RNA binding motif, single stranded interacting protein | 2.19 |
| TRIM32 | NM_012210 | Homo sapiens tripartite motif-containing 32 | 2.20 |
| PLCXD3 | NM_001005473 | Homo sapiens phosphatidylinositol-specific phospholipase C, X domain containing 3 | 2.20 |
| ZFP42 | NM_174900 | Homo sapiens zinc finger protein 42 | 2.20 |
| KNG1 | NM_000893 | Homo sapiens kininogen 1 | 2.20 |
| SCGB2A2 | NM_002411 | Homo sapiens secretoglobin, family 2A, member 2 | 2.20 |
| DLG3 | NM_021120 | Homo sapiens discs, large homolog 3 (neuroendocrine-dlg, Drosophila) | 2.21 |
| ONECUT2 | NM_004852 | Homo sapiens one cut domain, family member 2 | 2.21 |
| KITLG | NM_000899 | Homo sapiens KIT ligand | 2.21 |
| MLPH | NM_024101 | Homo sapiens melanophilin | 2.21 |
| NPPC | NM_024409 | Homo sapiens natriuretic peptide precursor C | 2.21 |
| SEL1L | NM_005065 | Homo sapiens sel-1 suppressor of lin-12-like (C. elegans) | 2.21 |
| ETEA | NM_014613 | Homo sapiens expressed in T-cells and eosinophils in atopic dermatitis | 2.22 |
| VGLL4 | NM_014667 | Homo sapiens vestigial like 4 (Drosophila) | 2.22 |
| PLCB2 | NM_004573 | Homo sapiens phospholipase C, beta 2 | 2.22 |
| LCN6 | NM_198946 | Homo sapiens lipocalin 6 | 2.22 |
| HBS1L | NM_006620 | Homo sapiens HBS1-like (S. cerevisiae) | 2.22 |
| PDE6A | NM_000440 | Homo sapiens phosphodiesterase 6A, cGMP-specific, rod, alpha | 2.23 |
| KLF12 | NM_016285 | Homo sapiens Kruppel-like factor 12 | 2.23 |
| BBOX1 | NM_003986 | Homo sapiens butyrobetaine (gamma), 2-oxoglutarate dioxygenase (gamma-butyrobetaine hydroxylase) 1 | 2.23 |
| CNGA3 | NM_001298 | Homo sapiens cyclic nucleotide gated channel alpha 3 | 2.23 |
| COL5A2 | NM_000393 | Homo sapiens collagen, type V, alpha 2 | 2.24 |
| DGKB | NM_004080 | Homo sapiens diacylglycerol kinase, beta 90kDa | 2.24 |
| PAMCI | NM_005447 | Homo sapiens peptidylglycine alpha-amidating monooxygenase COOH-terminal interactor | 2.24 |
| ARPP-21 | NM_198399 | Homo sapiens cyclic AMP-regulated phosphoprotein, 21 kDa | 2.24 |
| A_24_P919812 | | Unknown | 2.24 |
| ATP11B | NM_014616 | Homo sapiens ATPase, Class VI, type 11B | 2.24 |
| GSTM3 | NM_000849 | Homo sapiens glutathione S-transferase M3 (brain) | 2.25 |
| NEK2 | NM_002497 | Homo sapiens NIMA (never in mitosis gene a)-related kinase 2 | 2.25 |
| SDC4 | NM_002999 | Homo sapiens syndecan 4 | 2.25 |
| SYNJ2 | NM_003898 | Homo sapiens synaptojanin 2 | 2.25 |
| GCM2 | NM_004752 | Homo sapiens glial cells missing homolog 2 | 2.26 |
| NBR2 | NM_005821 | Homo sapiens neighbor of BRCA1 gene 2 | 2.26 |
| TRPM6 | NM_017662 | Homo sapiens transient receptor potential cation channel, subfamily M, member 6 | 2.27 |
| IL22 | NM_020525 | Homo sapiens interleukin 22 | 2.27 |
| SLITRK3 | NM_014926 | Homo sapiens SLIT and NTRK-like family, member 3 | 2.27 |
| APOBEC1 | NM_001644 | Homo sapiens apolipoprotein B mRNA editing enzyme, catalytic polypeptide 1 | 2.27 |
| TRIM40 | NM_138700 | Homo sapiens tripartite motif-containing 40 | 2.27 |
| CKAP2 | NM_018204 | Homo sapiens cytoskeleton associated protein 2 | 2.28 |
| CSPG4 | NM_001897 | Homo sapiens chondroitin sulfate proteoglycan 4 (melanoma-associated) | 2.28 |
| DSC3 | NM_024423 | Homo sapiens desmocollin 3 | 2.28 |
| FAM9C | NM_174901 | Homo sapiens family with sequence similarity 9, member C | 2.28 |
| WDR49 | NM_178824 | Homo sapiens WD repeat domain 49 | 2.29 |
| RAG1 | NM_000448 | Homo sapiens recombination activating gene 1 | 2.29 |
| ANKRD25 | NM_015493 | Homo sapiens ankyrin repeat domain 25 | 2.29 |
| MOBP | NM_006501 | Homo sapiens myelin-associated oligodendrocyte basic protein | 2.29 |
| IVL | NM_005547 | Homo sapiens involucrin | 2.29 |
| RHBDL2 | NM_017821 | Homo sapiens rhomboid, veinlet-like 2 | 2.30 |
| CLUL1 | NM_014410 | Homo sapiens clusterin-like 1 (retinal) | 2.30 |
| COL4A4 | NM_000092 | Homo sapiens collagen, type IV, alpha 4 | 2.30 |
| WFIKKN2 | NM_175575 | Homo sapiens WAP, follistatin/kazal, immunoglobulin, kunitz and netrin domain containing 2 | 2.30 |
| DAND5 | NM_152654 | Homo sapiens DAN domain family, member 5 | 2.31 |
| SPTBN1 | NM_178313 | Homo sapiens spectrin, beta, non-erythrocytic 1 | 2.31 |
| LRP11 | NM_032832 | Homo sapiens low density lipoprotein receptor-related protein 11 | 2.31 |
| KLHL14 | NM_020805 | Homo sapiens kelch-like 14 (Drosophila) | 2.31 |
| TEX15 | NM_031271 | Homo sapiens testis expressed sequence 15 | 2.32 |
| FIGN | NM_018086 | Homo sapiens fidgetin | 2.32 |
| CDH22 | NM_021248 | Homo sapiens cadherin-like 22 | 2.32 |
| PDZK10 | NM_014728 | Homo sapiens PDZ domain containing 10 | 2.33 |
| MIA2 | NM_054024 | Homo sapiens melanoma inhibitory activity 2 | 2.33 |
| ROPN1L | NM_031916 | Homo sapiens ropporin 1-like | 2.33 |
| IQCA | NM_024726 | Homo sapiens IQ motif containing with AAA domain | 2.33 |
| ARSJ | NM_024590 | Homo sapiens arylsulfatase J | 2.34 |
| NALP6 | NM_138329 | Homo sapiens NACHT, leucine rich repeat and PYD containing 6 | 2.34 |
| ALB | NM_000477 | Homo sapiens albumin | 2.34 |
| FGB | NM_005141 | Homo sapiens fibrinogen, B beta polypeptide | 2.34 |
| NM_001017967 | NM_001017967 | Homo sapiens MARVEL domain containing 3 | 2.34 |
| L1CAM | NM_024003 | Homo sapiens L1 cell adhesion molecule | 2.34 |
| COLEC12 | NM_030781 | Homo sapiens collectin sub-family member 12 | 2.35 |
| NM_007257 | NM_007257 | Homo sapiens paraneoplastic antigen MA2 | 2.35 |
| VCAM1 | NM_001078 | Homo sapiens vascular cell adhesion molecule 1 | 2.35 |
| SLC9A11 | NM_178527 | Homo sapiens solute carrier family 9, isoform 11 | 2.35 |
| SLC39A14 | NM_015359 | Homo sapiens solute carrier family 39 (zinc transporter), member 14 | 2.35 |
| SLC5A8 | NM_145913 | Homo sapiens solute carrier family 5 (iodide transporter), member 8 | 2.35 |
| ALS2 | NM_020919 | Homo sapiens amyotrophic lateral sclerosis 2 | 2.35 |
| HECW1 | NM_015052 | Homo sapiens HECT, C2 and WW domain containing E3 ubiquitin protein ligase 1 | 2.35 |
| HIST1H1B | NM_005322 | Homo sapiens histone 1, H1b | 2.35 |
| CCL16 | NM_004590 | Homo sapiens chemokine (C-C motif) ligand 16 | 2.36 |
| GFAP | NM_002055 | Homo sapiens glial fibrillary acidic protein | 2.36 |
| TCF19 | NM_007109 | Homo sapiens transcription factor 19 | 2.36 |
| UBAP2L | NM_014847 | Homo sapiens ubiquitin associated protein 2-like | 2.37 |
| SCAND2 | NM_033633 | Homo sapiens SCAN domain containing 2 | 2.37 |
| RGS13 | NM_002927 | Homo sapiens regulator of G-protein signalling 13 | 2.37 |
| KRT25B | NM_181539 | Homo sapiens keratin 25B | 2.37 |
| PSCA | NM_005672 | Homo sapiens prostate stem cell antigen | 2.37 |
| OGDH | NM_002541 | Homo sapiens oxoglutarate (alpha-ketoglutarate) dehydrogenase (lipoamide) | 2.38 |
| SLC12A5 | NM_020708 | Homo sapiens solute carrier family 12, (potassium-chloride transporter) member 5 | 2.38 |
| BMP3 | NM_001201 | Homo sapiens bone morphogenetic protein 3 (osteogenic) | 2.38 |
| FOXP2 | NM_148900 | Homo sapiens forkhead box P2 | 2.38 |
| SIGLEC11 | NM_052884 | Homo sapiens sialic acid binding Ig-like lectin 11 | 2.39 |
| MEP1B | NM_005925 | Homo sapiens meprin A, beta | 2.39 |
| TRAR3 | NM_175057 | Homo sapiens trace amine associated receptor 9 | 2.39 |
| RGS7 | NM_002924 | Homo sapiens regulator of G-protein signalling 7 | 2.39 |
| CBLC | NM_012116 | Homo sapiens Cas-Br-M (murine) ecotropic retroviral transforming sequence c | 2.39 |
| CCR8 | NM_005201 | Homo sapiens chemokine (C-C motif) receptor 8 | 2.39 |
| FBXO36 | NM_174899 | Homo sapiens F-box protein 36 | 2.39 |
| CENTB5 | NM_030649 | Homo sapiens centaurin, beta 5 (CENTB5), mRNA [NM_030649] | 2.40 |
| OMG | NM_002544 | Homo sapiens oligodendrocyte myelin glycoprotein | 2.40 |
| TPM2 | NM_213674 | Homo sapiens tropomyosin 2 (beta) | 2.40 |
| ROR1 | NM_005012 | Homo sapiens receptor tyrosine kinase-like orphan receptor 1 | 2.41 |
| TRPM1 | NM_002420 | Homo sapiens transient receptor potential cation channel, subfamily M, member 1 | 2.41 |
| SPANXB2 | NM_145664 | Homo sapiens SPANX family, member B2 | 2.41 |
| PCDHB10 | NM_018930 | Homo sapiens protocadherin beta 10 | 2.41 |
| IL20 | NM_018724 | Homo sapiens interleukin 20 | 2.41 |
| OXCT2 | NM_022120 | Homo sapiens 3-oxoacid CoA transferase 2 | 2.41 |
| TRIM15 | NM_033229 | Homo sapiens tripartite motif-containing 15 | 2.42 |
| IL1RAPL1 | NM_014271 | Homo sapiens interleukin 1 receptor accessory protein-like 1 | 2.42 |
| RALGPS2 | NM_152663 | Homo sapiens Ral GEF with PH domain and SH3 binding motif 2 | 2.42 |
| PLAC4 | NM_182832 | Homo sapiens placenta-specific 4 | 2.43 |
| HOXA5 | NM_019102 | Homo sapiens homeo box A5 | 2.43 |
| ADAMTSL1 | NM_139264 | Homo sapiens ADAMTS-like 1 | 2.44 |
| CSTF2T | NM_015235 | Homo sapiens cleavage stimulation factor, 3' pre-RNA, subunit 2, 64kDa, tau variant | 2.44 |
| FOXA1 | NM_004496 | Homo sapiens forkhead box A1 | 2.44 |
| SYNE2 | NM_182910 | Homo sapiens spectrin repeat containing, nuclear envelope 2 | 2.44 |
| TBCD | NM_005993 | Homo sapiens tubulin-specific chaperone d | 2.45 |
| EPHA1 | NM_005232 | Homo sapiens EPH receptor A1 | 2.45 |
| AF220235 | AF220235 | Homo sapiens FGF-2 activity-associated protein 3 | 2.46 |
| GTSE1 | NM_016426 | Homo sapiens G-2 and S-phase expressed 1 | 2.46 |
| SLC4A10 | NM_022058 | Homo sapiens solute carrier family 4, sodium bicarbonate transporter-like, member 10 | 2.46 |
| ZNF44 | BC032246 | Homo sapiens zinc finger protein 44 | 2.46 |
| ZNF311 | NM_001010877 | Homo sapiens zinc finger protein 311 | 2.47 |
| CNTNAP4 | NM_138994 | Homo sapiens contactin associated protein-like 4 | 2.48 |
| EVE1 | NM_001009555 | Homo sapiens SH3 domain protein D19 | 2.49 |
| GKAP1 | NM_025211 | Homo sapiens G kinase anchoring protein 1 | 2.49 |
| RFX4 | NM_002920 | Homo sapiens regulatory factor X, 4 (influences HLA class II expression) | 2.49 |
| GRID1 | NM_017551 | Homo sapiens glutamate receptor, ionotropic, delta 1 | 2.49 |
| PTPRS | NM_130853 | Homo sapiens protein tyrosine phosphatase, receptor type, S | 2.50 |
| DTNB | NM_033148 | Homo sapiens dystrobrevin, beta | 2.50 |
| KIRREL2 | NM_199180 | Homo sapiens kin of IRRE like 2 (Drosophila) | 2.50 |
| OTEX | NM_139282 | Homo sapiens paired-like homeobox protein OTEX | 2.50 |
| NALP12 | NM_033297 | Homo sapiens NACHT, leucine rich repeat and PYD containing 12 | 2.51 |
| SUPT3H | NM_003599 | Homo sapiens suppressor of Ty 3 homolog | 2.51 |
| PLA2G2F | NM_022819 | Homo sapiens phospholipase A2, group IIF | 2.51 |
| AMELY | NM_001143 | Homo sapiens amelogenin, Y-linked | 2.53 |
| CGB1 | NM_033377 | Homo sapiens chorionic gonadotropin, beta polypeptide 1 | 2.53 |
| HOZFP | NM_152995 | Homo sapiens nuclear transcription factor, X-box binding-like 1 | 2.54 |
| SDK1 | NM_152744 | Homo sapiens sidekick homolog 1 (chicken) (SDK1), mRNA [NM_152744] | 2.54 |
| ALS2CR14 | NM_178231 | Homo sapiens amyotrophic lateral sclerosis 2 (juvenile) chromosome region, candidate 14 | 2.54 |
| GNGT1 | NM_021955 | Homo sapiens guanine nucleotide binding protein (G protein), gamma transducing activity polypeptide 1 | 2.54 |
| SERPINA5 | NM_000624 | Homo sapiens serine (or cysteine) proteinase inhibitor, clade A (alpha-1 antiproteinase, antitrypsin), member 5 | 2.54 |
| CYP8B1 | NM_004391 | Homo sapiens cytochrome P450, family 8, subfamily B, polypeptide 1 | 2.54 |
| MAGEA4 | NM_002362 | Homo sapiens melanoma antigen family A, 4 | 2.55 |
| TNNC2 | NM_003279 | Homo sapiens troponin C2, fast | 2.55 |
| APOF | NM_001638 | Homo sapiens apolipoprotein F | 2.55 |
| RAP1GA1 | NM_002885 | Homo sapiens RAP1, GTPase activating protein 1 | 2.55 |
| IL7 | NM_000880 | Homo sapiens interleukin 7 | 2.56 |
| ENAH | NM_001008493 | Homo sapiens enabled homolog (Drosophila) | 2.56 |
| CCNE2 | NM_057749 | Homo sapiens cyclin E2 | 2.56 |
| ID4 | NM_001546 | Homo sapiens inhibitor of DNA binding 4, dominant negative helix-loop-helix protein | 2.56 |
| PRKG1 | NM_006258 | Homo sapiens protein kinase, cGMP-dependent, type I | 2.57 |
| TSKS | NM_021733 | Homo sapiens testis-specific kinase substrate | 2.57 |
| CASR | NM_000388 | Homo sapiens calcium-sensing receptor | 2.57 |
| FBXW10 | NM_031456 | Homo sapiens F-box and WD-40 domain protein 10 | 2.57 |
| GJA7 | NM_005497 | Homo sapiens gap junction protein, alpha 7, 45kDa (connexin 45) | 2.58 |
| KCNQ4 | NM_004700 | Homo sapiens potassium voltage-gated channel, KQT-like subfamily, member 4 | 2.58 |
| L1CAM | NM_000425 | Homo sapiens L1 cell adhesion molecule | 2.59 |
| FOSL1 | NM_005438 | Homo sapiens FOS-like antigen 1 | 2.59 |
| MYO15A | NM_016239 | Homo sapiens myosin XVA | 2.59 |
| FGF22 | NM_020637 | Homo sapiens fibroblast growth factor 22 | 2.59 |
| EPHB1 | NM_004441 | Homo sapiens EPH receptor B1 | 2.59 |
| EFTUD1 | NM_024580 | Homo sapiens elongation factor Tu GTP binding domain containing 1 | 2.60 |
| KCNS1 | NM_002251 | Homo sapiens potassium voltage-gated channel, delayed-rectifier, subfamily S, member 1 | 2.60 |
| OCLN | NM_002538 | Homo sapiens occludin | 2.62 |
| KBTBD10 | NM_006063 | Homo sapiens kelch repeat and BTB (POZ) domain containing 10 | 2.62 |
| GARNL3 | NM_032293 | Homo sapiens GTPase activating Rap/RanGAP domain-like 3 | 2.62 |
| MAS1 | NM_002377 | Homo sapiens MAS1 oncogene | 2.62 |
| IFNK | NM_020124 | Homo sapiens interferon, kappa | 2.64 |
| SLC25A23 | NM_024103 | Homo sapiens solute carrier family 25 (mitochondrial carrier; phosphate carrier), member 23 | 2.65 |
| Cep70 | NM_024491 | Homo sapiens p10-binding protein | 2.66 |
| FGF5 | NM_004464 | Homo sapiens fibroblast growth factor 5 | 2.67 |
| RAPGEF3 | NM_006105 | Homo sapiens Rap guanine nucleotide exchange factor (GEF) 3 | 2.67 |
| TZFP | NM_014383 | Homo sapiens testis zinc finger protein | 2.68 |
| ANGPTL3 | NM_014495 | Homo sapiens angiopoietin-like 3 | 2.69 |
| GPAM | NM_020918 | Homo sapiens glycerol-3-phosphate acyltransferase, mitochondrial | 2.69 |
| CER1 | NM_005454 | Homo sapiens cerberus 1 homolog, cysteine knot superfamily (Xenopus laevis) | 2.70 |
| NDRG2 | NM_201535 | Homo sapiens NDRG family member 2 | 2.70 |
| GAD1 | NM_000817 | Homo sapiens glutamate decarboxylase 1 (brain, 67kDa) | 2.71 |
| MMP20 | NM_004771 | Homo sapiens matrix metalloproteinase 20 (enamelysin) | 2.72 |
| XLKD1 | NM_006691 | Homo sapiens extracellular link domain containing 1 | 2.72 |
| IGFBP5 | NM_000599 | Homo sapiens insulin-like growth factor binding protein 5 | 2.74 |
| MAN1C1 | NM_020379 | Homo sapiens mannosidase, alpha, class 1C, member 1 | 2.74 |
| MYH6 | NM_002471 | Homo sapiens myosin, heavy polypeptide 6, cardiac muscle, alpha (cardiomyopathy, hypertrophic 1) | 2.74 |
| KLK11 | NM_144947 | Homo sapiens kallikrein 11 | 2.75 |
| ADAM30 | NM_021794 | Homo sapiens a disintegrin and metalloproteinase domain 30 | 2.75 |
| CLEC4M | NM_214675 | Homo sapiens C-type lectin domain family 4, member M | 2.75 |
| ATF7IP2 | NM_024997 | Homo sapiens activating transcription factor 7 interacting protein 2 | 2.75 |
| CDO1 | NM_001801 | Homo sapiens cysteine dioxygenase, type I | 2.76 |
| FGA | NM_021871 | Homo sapiens fibrinogen, A alpha polypeptide | 2.76 |
| PFN2 | NM_053024 | Homo sapiens profilin 2 | 2.76 |
| COX7B2 | NM_130902 | Homo sapiens cytochrome c oxidase subunit VIIb2 | 2.76 |
| SOX6 | NM_033326 | Homo sapiens SRY (sex determining region Y)-box 6 | 2.79 |
| RBP2 | NM_004164 | Homo sapiens retinol binding protein 2, cellular | 2.79 |
| TU3A | NM_007177 | Homo sapiens TU3A protein | 2.80 |
| GPR110 | NM_025048 | Homo sapiens G protein-coupled receptor 110 | 2.80 |
| NKX2-3 | NM_145285 | Homo sapiens NK2 transcription factor related, locus 3 (Drosophila) | 2.81 |
| STMN2 | NM_007029 | Homo sapiens stathmin-like 2 | 2.82 |
| GSTM5 | NM_000851 | Homo sapiens glutathione S-transferase M5 | 2.82 |
| OLFM1 | NM_014279 | Homo sapiens olfactomedin 1 | 2.83 |
| SCN3A | NM_006922 | Homo sapiens sodium channel, voltage-gated, type III, alpha | 2.83 |
| ESRRG | NM_206594 | Homo sapiens estrogen-related receptor gamma | 2.84 |
| SCN7A | NM_002976 | Homo sapiens sodium channel, voltage-gated, type VII, alpha | 2.85 |
| PSD | NM_002779 | Homo sapiens pleckstrin and Sec7 domain containing | 2.85 |
| RNF128 | NM_194463 | Homo sapiens ring finger protein 128 | 2.86 |
| HIST1H1A | NM_005325 | Homo sapiens histone 1, H1a | 2.86 |
| PLK4 | NM_014264 | Homo sapiens polo-like kinase 4 (Drosophila) | 2.88 |
| MATN2 | NM_030583 | Homo sapiens matrilin 2 | 2.88 |
| STK33 | NM_030906 | Homo sapiens serine/threonine kinase 33 | 2.89 |
| RCV1 | NM_002903 | Homo sapiens recoverin | 2.91 |
| KIF9 | NM_022342 | Homo sapiens kinesin family member 9 | 2.92 |
| FLT1 | NM_002019 | Homo sapiens fms-related tyrosine kinase 1 (vascular endothelial growth factor/vascular permeability factor receptor) | 2.92 |
| SEC14L2 | NM_012429 | Homo sapiens SEC14-like 2 | 2.93 |
| EPHA8 | NM_001006943 | Homo sapiens EPH receptor A8 | 2.95 |
| GRPR | NM_005314 | Homo sapiens gastrin-releasing peptide receptor | 2.95 |
| KHDRBS2 | NM_152688 | Homo sapiens KH domain containing, RNA binding, signal transduction associated 2 | 2.96 |
| GMCL1L | NM_022471 | Homo sapiens germ cell-less homolog 1 (Drosophila)-like | 2.96 |
| ARGBP2 | NM_021069 | Homo sapiens Arg/Abl-interacting protein ArgBP2 | 2.96 |
| KIF12 | NM_138424 | Homo sapiens kinesin family member 12 | 2.96 |
| PTH | NM_000315 | Homo sapiens parathyroid hormone | 2.96 |
| HHLA3 | NM_007071 | Homo sapiens HERV-H LTR-associating 3 | 2.99 |
| GJA8 | NM_005267 | Homo sapiens gap junction protein, alpha 8, 50kDa (connexin 50) | 3.00 |
| SLC25A21 | NM_030631 | Homo sapiens solute carrier family 25 | 3.01 |
| ZNF610 | NM_173530 | Homo sapiens zinc finger protein 610 | 3.02 |
| ADAM2 | NM_001464 | Homo sapiens a disintegrin and metalloproteinase domain 2 | 3.02 |
| GNG3 | NM_012202 | Homo sapiens guanine nucleotide binding protein (G protein), gamma 3 | 3.04 |
| FAM47B | NM_152631 | Homo sapiens family with sequence similarity 47, member B | 3.05 |
| ARHGEF10 | NM_014629 | Homo sapiens Rho guanine nucleotide exchange factor (GEF) 10 | 3.05 |
| HEY2 | NM_012259 | Homo sapiens hairy/enhancer-of-split related with YRPW motif 2 | 3.06 |
| HOXA13 | NM_000522 | Homo sapiens homeo box A13 | 3.06 |
| FCAR | NM_133280 | Homo sapiens Fc fragment of IgA, receptor for | 3.07 |
| RAB17 | NM_022449 | Homo sapiens RAB17, member RAS oncogene family | 3.07 |
| SPANXA1 | NM_013453 | Homo sapiens sperm protein associated with the nucleus, X-linked, family member A1 | 3.07 |
| GPR83 | NM_016540 | Homo sapiens G protein-coupled receptor 83 | 3.08 |
| FSIP2 | NM_173651 | Homo sapiens fibrous sheath interacting protein 2 | 3.09 |
| ZIC1 | NM_003412 | Homo sapiens Zic family member 1 (odd-paired homolog, Drosophila) | 3.10 |
| ADAMTSL3 | NM_207517 | Homo sapiens ADAMTS-like 3 | 3.10 |
| VAMP1 | NM_016830 | Homo sapiens vesicle-associated membrane protein 1 (synaptobrevin 1) | 3.10 |
| ASAM | NM_024769 | Homo sapiens adipocyte-specific adhesion molecule | 3.11 |
| LGI2 | NM_018176 | Homo sapiens leucine-rich repeat LGI family, member 2 | 3.11 |
| AGXT2 | NM_031900 | Homo sapiens alanine-glyoxylate aminotransferase 2 (AGXT2), nuclear gene encoding mitochondrial protein | 3.12 |
| DMD | NM_004019 | Homo sapiens dystrophin (muscular dystrophy, Duchenne and Becker types) | 3.12 |
| AKAP4 | NM_003886 | Homo sapiens A kinase (PRKA) anchor protein 4 | 3.13 |
| ANGPTL1 | NM_004673 | Homo sapiens angiopoietin-like 1 | 3.14 |
| LCT | NM_002299 | Homo sapiens lactase | 3.14 |
| SYNJ2 | NM_003898 | Homo sapiens synaptojanin 2 | 3.15 |
| GATA5 | NM_080473 | Homo sapiens GATA binding protein 5 | 3.16 |
| GDF10 | NM_004962 | Homo sapiens growth differentiation factor 10 | 3.16 |
| GPR4 | NM_005282 | Homo sapiens G protein-coupled receptor 4 | 3.17 |
| NR3C2 | NM_000901 | Homo sapiens nuclear receptor subfamily 3, group C, member 2 | 3.18 |
| SCML4 | NM_198081 | Homo sapiens sex comb on midleg-like 4 (Drosophila) | 3.26 |
| UPK2 | NM_006760 | Homo sapiens uroplakin 2 | 3.26 |
| FBP2 | NM_003837 | Homo sapiens fructose-1,6-bisphosphatase 2 | 3.28 |
| IL17E | NM_022789 | Homo sapiens interleukin 17E | 3.32 |
| DLEC1 | NM_007338 | Homo sapiens deleted in lung and esophageal cancer 1 (DLEC1), transcript variant DLEC1-L1 | 3.36 |
| PRSS12 | NM_003619 | Homo sapiens protease, serine, 12 (neurotrypsin, motopsin) | 3.40 |
| RFPL3 | NM_006604 | Homo sapiens ret finger protein-like 3 | 3.41 |
| MASP1 | NM_139125 | Homo sapiens mannan-binding lectin serine protease 1 (C4/C2 activating component of Ra-reactive factor) | 3.41 |
| DOK6 | NM_152721 | Homo sapiens docking protein 6 | 3.42 |
| SH3BGRL2 | NM_031469 | Homo sapiens SH3 domain binding glutamic acid-rich protein like 2 | 3.44 |
| EDA | NM_001005614 | Homo sapiens ectodysplasin A | 3.48 |
| MYR8 | NM_015011 | Homo sapiens myosin heavy chain Myr 8 | 3.51 |
| FAM19A3 | NM_001004440 | Homo sapiens family with sequence similarity 19 (chemokine (C-C motif)-like), member A3 | 3.56 |
| MIG-6 | NM_018948 | Homo sapiens Gene 33\/Mig-6 (MIG-6) | 3.58 |
| COL19A1 | NM_001858 | Homo sapiens collagen, type XIX, alpha 1 | 3.58 |
| ANXA10 | NM_007193 | Homo sapiens annexin A10 | 3.63 |
| TTID | NM_006790 | Homo sapiens titin immunoglobulin domain protein (myotilin) | 3.65 |
| TCF15 | NM_004609 | Homo sapiens transcription factor 15 (basic helix-loop-helix) | 3.73 |
| XPR1 | NM_004736 | Homo sapiens xenotropic and polytropic retrovirus receptor | 3.74 |
| HK1 | NM_033497 | Homo sapiens hexokinase 1 | 3.82 |
| PDE4D | NM_006203 | Homo sapiens phosphodiesterase 4D, cAMP-specific (phosphodiesterase E3 dunce homolog, Drosophila)] | 3.84 |
| NLGN4Y | NM_014893 | Homo sapiens neuroligin 4, Y-linked | 3.85 |
| IGFBP3 | NM_001013398 | Homo sapiens insulin-like growth factor binding protein 3 | 3.87 |
| TFCP2L3 | NM_024915 | Homo sapiens transcription factor CP2-like 3 | 3.89 |
| IGFL2 | NM_001002915 | Homo sapiens insulin growth factor-like family member 2 | 3.96 |
| AGTR1 | NM_031850 | Homo sapiens angiotensin II receptor, type 1 | 3.99 |
| CKMT1 | NM_020990 | Homo sapiens creatine kinase, mitochondrial 1 (ubiquitous) | 3.99 |
| PDGFRA | BC015186 | Homo sapiens platelet-derived growth factor receptor, alpha polypeptide | 3.99 |
| P4HA3 | NM_182904 | Homo sapiens procollagen-proline, 2-oxoglutarate 4-dioxygenase (proline 4-hydroxylase), alpha polypeptide III | 3.99 |
| SPAM1 | NM_153189 | Homo sapiens sperm adhesion molecule 1 (PH-20 hyaluronidase, zona pellucida binding) | 4.02 |
| SYCP1 | NM_003176 | Homo sapiens synaptonemal complex protein 1 | 4.06 |
| ISL1 | NM_002202 | Homo sapiens ISL1 transcription factor, LIM/homeodomain, (islet-1) | 4.06 |
| CLIC6 | NM_053277 | Homo sapiens chloride intracellular channel 6 | 4.08 |
| PCDH18 | NM_019035 | Homo sapiens protocadherin 18 | 4.10 |
| EDG8 | NM_030760 | Homo sapiens endothelial differentiation, sphingolipid G-protein-coupled receptor, 8 | 4.12 |
| KSP37 | NM_031950 | Homo sapiens Ksp37 protein | 4.19 |
| LRRC2 | NM_024512 | Homo sapiens leucine rich repeat containing 2 | 4.20 |
| SCUBE2 | NM_020974 | Homo sapiens signal peptide, CUB domain, EGF-like 2 | 4.23 |
| CMYA5 | NM_153610 | Homo sapiens cardiomyopathy associated 5 | 4.24 |
| PCDHB16 | NM_020957 | Homo sapiens protocadherin beta 16 | 4.30 |
| GDPD2 | NM_017711 | Homo sapiens glycerophosphodiester phosphodiesterase domain containing 2 | 4.35 |
| PPM1E | NM_014906 | Homo sapiens protein phosphatase 1E (PP2C domain containing) | 4.35 |
| PTPN13 | NM_080685 | Homo sapiens protein tyrosine phosphatase, non-receptor type 13 (APO-1/CD95 (Fas)-associated phosphatase) | 4.35 |
| ULK4 | BC040739 | Homo sapiens unc-51-like kinase 4 (C. elegans) | 4.45 |
| CNTN4 | NM_175607 | Homo sapiens contactin 4 | 4.46 |
| PGAP1 | NM_024989 | Homo sapiens GPI deacylase | 4.48 |
| BTNL9 | NM_152547 | Homo sapiens butyrophilin-like 9 | 4.52 |
| CTTNBP2 | NM_033427 | Homo sapiens cortactin binding protein 2 | 4.52 |
| SOCS7 | NM_014598 | Homo sapiens suppressor of cytokine signaling 7 | 4.52 |
| ALF | NM_172196 | Homo sapiens TFIIA-alpha/beta-like factor | 4.60 |
| SLAMF6 | NM_052931 | Homo sapiens SLAM family member 6 | 4.62 |
| OLFM1 | NM_006334 | Homo sapiens olfactomedin 1 | 4.69 |
| ANGPT2 | NM_001147 | Homo sapiens angiopoietin 2 | 4.72 |
| ABCB5 | NM_178559 | Homo sapiens ATP-binding cassette, sub-family B (MDR/TAP), member 5 | 4.79 |
| ZNF501 | NM_145044 | Homo sapiens zinc finger protein 501 | 4.85 |
| SOX5 | NM_152989 | Homo sapiens SRY (sex determining region Y)-box 5 | 4.85 |
| PBX1 | NM_002585 | Homo sapiens pre-B-cell leukemia transcription factor 1 | 4.88 |
| RBMY1B | NM_001006121 | Homo sapiens RNA binding motif protein, Y-linked, family 1, member B | 4.94 |
| PSG9 | NM_002784 | Homo sapiens pregnancy specific beta-1-glycoprotein 9 | 4.95 |
| CABYR | NM_012189 | Homo sapiens calcium-binding tyrosine-(Y)-phosphorylation regulated (fibrousheathin 2) | 5.05 |
| SLITRK1 | NM_052910 | Homo sapiens SLIT and NTRK-like family, member 1 | 5.17 |
| PROZ | NM_003891 | Homo sapiens protein Z, vitamin K-dependent plasma glycoprotein | 5.38 |
| PDZRN4 | NM_013377 | Homo sapiens PDZ domain containing RING finger 4 | 5.60 |
| STARD6 | NM_139171 | Homo sapiens START domain containing 6 | 5.61 |
| CKLFSF1 | NM_052999 | Homo sapiens chemokine-like factor super family 1 | 5.74 |
| ESPN | NM_031475 | Homo sapiens espin | 5.88 |
| HOXC10 | NM_017409 | Homo sapiens homeo box C10 | 5.94 |
| EPB41L5 | BC032822 | Homo sapiens erythrocyte membrane protein band 4.1 like 5 | 6.02 |
| TSNAXIP1 | NM_018430 | Homo sapiens translin-associated factor X interacting protein 1 | 6.07 |
| ARHGAP28 | NM_001010000 | Homo sapiens Rho GTPase activating protein 28 | 6.38 |
| MOV10L1 | NM_018995 | Homo sapiens Mov10l1, Moloney leukemia virus 10-like 1, homolog (mouse) | 6.39 |
| FILIP1 | NM_015687 | Homo sapiens filamin A interacting protein 1 | 6.41 |
| PCDHB15 | NM_018935 | Homo sapiens protocadherin beta 15 | 7.25 |
| ESAM | NM_138961 | Homo sapiens endothelial cell adhesion molecule | 7.94 |
| TGFB3 | NM_003239 | Homo sapiens transforming growth factor, beta 3 | 10.89 |
| TIGD4 | NM_145720 | Homo sapiens tigger transposable element derived 4 | 11.02 |
